# The evolving battle between yellow rust and wheat: implications for global food security

**DOI:** 10.1007/s00122-021-03983-z

**Published:** 2021-11-25

**Authors:** Laura Bouvet, Sarah Holdgate, Lucy James, Jane Thomas, Ian J. Mackay, James Cockram

**Affiliations:** 1 John Bingham Laboratory, NIAB, 93 Lawrence Weaver Road, Cambridge, CB3 0LE UK; 2 Department of Plant Sciences, University of Cambridge, Downing Street, Cambridge, CB2 3EA UK; 3 Present Address: Scotland’s Rural College (SRUC), The King’s Buildings, West Mains Road, Edinburgh, EH9 3JG UK

## Abstract

Wheat (*Triticum aestivum* L.) is a global commodity, and its production is a key component underpinning worldwide food security. Yellow rust, also known as stripe rust, is a wheat disease caused by the fungus *Puccinia striiformis* Westend f. sp. *tritici* (*Pst*), and results in yield losses in most wheat growing areas. Recently, the rapid global spread of genetically diverse sexually derived *Pst* races, which have now largely replaced the previous clonally propagated slowly evolving endemic populations, has resulted in further challenges for the protection of global wheat yields. However, advances in the application of genomics approaches, in both the host and pathogen, combined with classical genetic approaches, pathogen and disease monitoring, provide resources to help increase the rate of genetic gain for yellow rust resistance via wheat breeding while reducing the carbon footprint of the crop. Here we review key elements in the evolving battle between the pathogen and host, with a focus on solutions to help protect future wheat production from this globally important disease.

Wheat (*Triticum aestivum* L.) is one of the most important staple crops, with global demand predicted to increase to 324 kg/year (per capita) by 2050 (Alexandratos and Bruinsma 2012). Wheat production faces numerous threats, with 10–16% of global wheat harvests estimated to be lost due to pests and diseases (Oerke 2006; Strange and Scott 2005). Yellow rust (YR), also known as stripe rust, is a major disease of wheat caused by the biotrophic fungal pathogen, *Puccinia striiformis* Westend f. sp. *tritici* (*Pst*). YR infection is most commonly noted on wheat leaves, where the resulting damage to photosynthetic tissues leads to reduced light interception and radiation use efficiency, thus lowering yields (Fig. 1a–b). However, YR infection can also take place on the structures of the wheat ear such as the glumes, lemma and palea, particularly during moderate to severe epidemics, resulting in reduced grain yield and quality (Bouvet et al. 2021a; Cromey 1989; Wellings, 2003; 2009) (Fig. 1c). Recurrent *Pst* epidemics have occurred in the majority of wheat growing areas over the past 60 years and can cause significant yield losses and reductions in grain quality if not adequately controlled (Wellings 2011). Notably, the past two decades have seen the rapid global emergence of more aggressive and genetically diverse *Pst* populations adapted to warmer temperatures (Hovmøller et al. 2016; Hubbard et al. 2015; Milus et al. 2009), with concomitant impact on the YR resistance ratings of many wheat varieties. YR resistance breeding targets have had to adapt to tackle the rapidly changing *Pst* threat, and sources of genetic resistance for the development of improved wheat varieties are continually being sought. This is now being aided by advances in wheat genomics approaches, as well as detailed characterisation of *Pst* population pathotypes, genetic diversity, effector characterisation and field monitoring. Ultimately, efficient control of wheat fungal disease will be via approaches that combine agricultural and agronomic practices, disease monitoring, and varietal genetic improvement (Downie et al. 2020). In this review, we summarise current understanding of the *Pst* lifecycle, modes of dispersal and genetic diversity, and wheat genetic resistance and highlight some of the challenges facing the efforts to maintain adequate protection against wheat YR infection, with a focus on genetic resistance approaches.

**Fig. 1:**
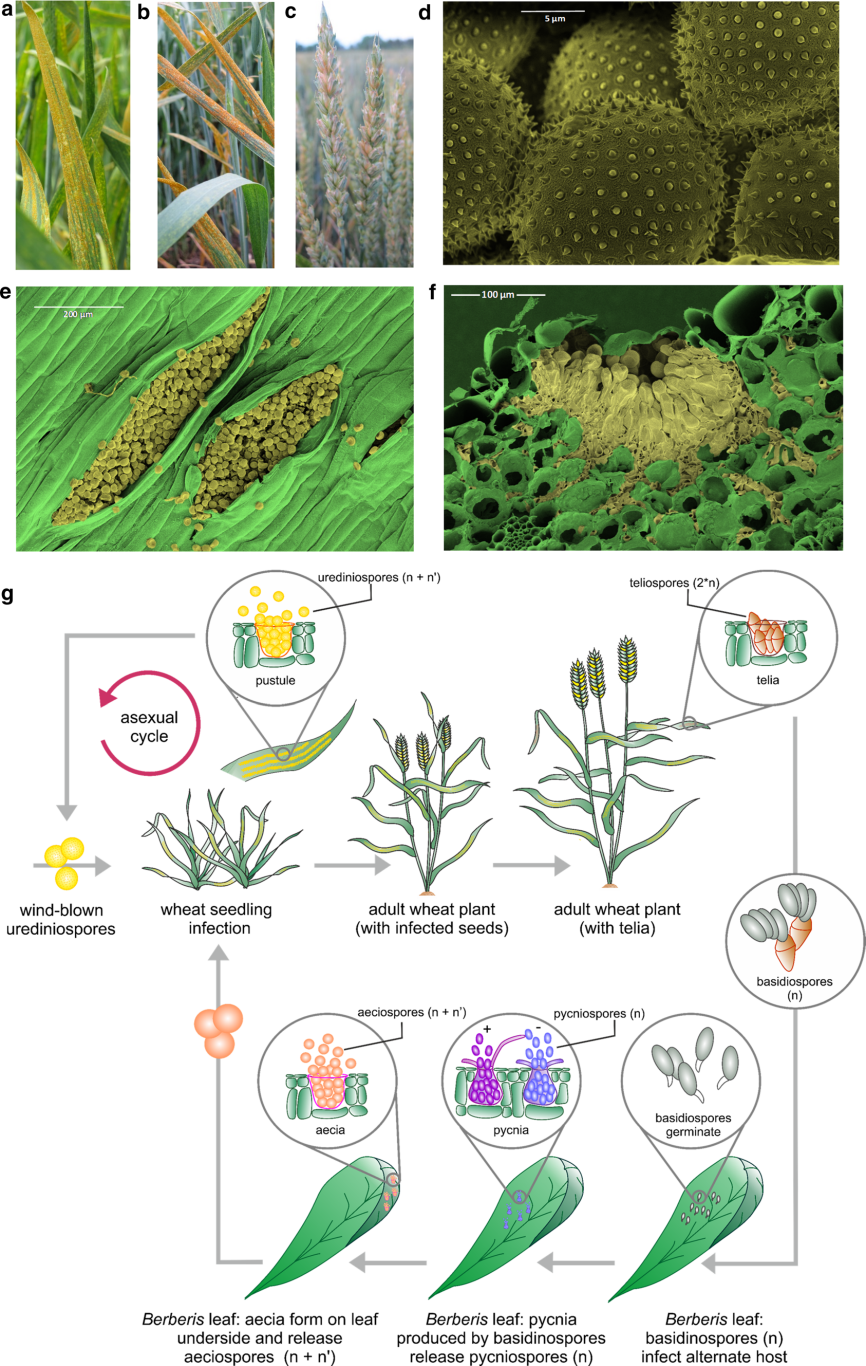
The *Puccinia striiformis* Westend f. sp. *tritici* (*Pst*) lifecycle. Wheat *Pst* infection at the adult plant stage in wheat, showing **a** yellow, and **b** orange coloured pustules that shed urediniospores. **c** Symptoms of *Pst* infection of the wheat ear. False colour scanning electron microscopy images of **d** urediniospores, **e** pustules on the wheat leaf surface, and **f** pustule in cross section. **g** Diagram illustrating the main features of the *Pst* lifecycle. Top left: Wheat plants can be infected by, (i) wind-blown single-cell dihaploid dikaryotic urediniospores (containing one haploid genome copy in each of the two nuclei within the cell: n + n′) produced on wheat, or (ii) by aeciospores (n + n′) produced on the alternative host (*Berberis* spp.). Yellow rust infection is typically observable on the heat upper leaf surface as parallel rows of yellow to orange pustules which release urediniospores, resulting in cycles of re-infection and cross-infection in wheat. Top middle: at ear emergence, yellow rust infection can occur on the florets of the wheat ear. Top right: towards the end of the wheat season, telia may form on the underside of the epidermis, from which diploid doubled haploid (2^*^n) two-celled teliospores are produced by karyogamy. Teliospores readily germinate to produce haploid basidiospores. Bottom right: Basidiospores germinate and infect leaves of the alternate host. Bottom middle: Basidiospore infection leads to the production of pycnia, typically on the upper side of the leaves, which release haploid pycniospores. Fusion of pycniospores with the receptive hypha of a mating-type compatible pycnia leads to dikaryotization and the development of aecia on the leaf underside. Bottom left: Aecia release vegetative aeciospores (n + n′) which are only able to infect the primary host species (predominantly wheat). For more information, see Chen et al. (2014), Schwessinger (2016) or Mehmood et al. (2020) (colour figure online)

## The complex pathogen lifecycle

*Pst* goes through five different spore stages and requires two plant host species for completion of its lifecycle (Fig. 1d). The two broad stages of the *Pst* lifecycle are classified as: (i) the asexual stage, which occurs on wheat (the primary host), and (ii) the sexual stage which occurs on *Berberis* species (the alternate host). In wheat, YR disease occurs during the asexual stage of the *Pst* lifecycle and is caused by multiple cycles of dikaryotic (i.e. two nuclei in each cell: n + n) *Pst* urediniospores re-infecting the primary host via wind dispersal. During the initial stage of wheat infection, urediniospores germinate on the leaf surface and eventually form an appressorium from which hyphae develop and enter the leaf tissue via the stomata. Growing hyphae develop into a dense network extending between and inside host mesophyll cells. Among this network, haustoria infection structures will form and specifically develop in host cell walls to extract nutrients (Szabo and Bushnell 2001). On the leaves of mature susceptible plants, disease symptoms are visible 12–14 days after infection, consisting of yellow to orange coloured urediniospores that erupt from pustules arranged in characteristic stripes that follow the veins down the leaf blade (Chen et al. 2014), which can lead to successive rounds of secondary infections. On resistant to mildly susceptible varieties, symptoms will range from non-sporulating flecks (a sign of hypersensitivity) to necrotic and chlorotic patches with no to limited sporulation. Towards the end of the wheat growth season, diploid teliospores may be produced by some isolates via karyogamy. These readily germinate to produce a promycelium of four cells, with meiosis subsequently resulting in a single haploid nucleus that forms a basidiospore able to infect the alternate host (Chen et al. 2014).

Although much less is known about the sexual stage, *Berberis* species were long speculated to support the *Pst* lifecycle (e.g. Straib 1937; Hart and Becker 1934), as well as the related rust species, *Puccinia graminis* f. sp. *tritici* (causal agent of stem rust). Historically this resulted in efforts to eradicate *Berberis* species in many European and North American countries (Barnes et al. 2020). However, it was not until 2010 that *Berberis* species were formally confirmed to support development of *Pst* pycnia and aecia (Jin et al. 2010). Curiously, *Berberis* species infected with *Pst* are rarely observed in the wild (Zhao et al. 2011, 2013). This may be due to the difficulty in finding an environment that simultaneously accommodates germination of teliospores (part of the asexual stage; enclosed in telia that form on wheat leaves towards the end of the infection season and produce basidiospores) and basidiospores (part of the sexual stage; forming on barberry leaves and requiring dew for germination), both of which have short viability (Wang and Chen 2015). A recent study showed *Berberis* species do not play a role in YR epidemics in the US Pacific Northwest (Wang and Chen 2015), and an additional alternate host, *Mahonia aquifolium* (Oregon grape) has been identified (Wang and Chen 2013). The main importance of the sexual *Pst* stage to wheat infection is the generation of novel combinations of standing genetic variation, resulting in new genetically recombined isolates that can cause widespread epidemics and rapid changes in wheat resistance profiles.

## Pathogens on the move: patterns of *Pst* dispersal and the rise of divergent lineages and aggressive races

Over the years, monitoring of virulence changes in *Pst* populations in the major wheat producing regions has revealed notable changes in pathogen movement and adaptation. These studies were based on pathogenicity surveys, which use sets of differential wheat lines carrying known resistance genes, either near isogenic lines or cultivars, for the characterisation of pathotypes at the seedling stage (Wellings et al. 2009). More recently, molecular and genomics techniques have been used to infer *Pst* population structure and genetic diversity, confirming patterns of adaptation hypothesised in pathotype-based approaches. Here, we summarise key findings and events from the past three decades, specifically focusing on patterns of spore dispersal and *Pst* evolution and adaptation.

### Blowing in the wind

*Pst* urediniospores are windborne and can disperse at continental scales. Coupled with the obligate nature of the pathogen (requiring living tissue to survive), this has led to different scenarios for the observed seasonal and geographic patterns of dispersal. One such model is the local extinction and re-colonisation model, illustrated for example in China where regions of the Sichuan and Gansu provinces in which *Pst* prevails all year round act as a source of inoculum to the more northerly provinces in which wheat is predominantly grown as a winter crop (Brown and Hovmøller 2002; Zeng and Luo 2006). In this way, *Pst* populations re-establish at the beginning of each wheat cropping season in those regions where *Pst* spores are usually unable to over-winter. A similar pattern of spore movement according to prevailing winds and the seasonality of the cropping seasons has been speculated in North America, with spores migrating from southern central states of USA and Mexico to northern central states of USA and Canada (Chen 2005). In North Western Europe, *Pst* spore dispersion appears to follow the continental-island model, first described by Hedrick (1985), and has been the predominant model of *Pst* spore dispersion in North Western Europe. In this region, urediniospores travel up to 1700 km with prevailing winds, and migrating between UK, France, Germany and Denmark (Hovmøller et al. 2002). Investigations of YR emergence events in countries where it was previously absent provide examples of rapid inter-continental foreign incursion. Australia has been subject to several known incursions, of which two were notably detrimental to the wheat industry due to their rapid spread: (i) the first occurrence of *Pst*, in 1979 (Wellings et al. 1987), and (ii) the 2002 incursion in Western Australia (Wellings et al. 2003), now known to have originated from the Middle East/East Africa (Ali et al. 2014a) and attributed to a single *Pst* isolate (Wellings et al. 2003). The more recent arrival of *Pst* isolates in South Africa in 1996 were related to the Mediterranean and Central Asian populations (Boshoff et al. 2002; Ali et al. 2014a), and was speculated to be due to wind dispersal or human activities (Ali et al. 2014a). In all three cases, human activity, most likely through accidental transport on clothing, has been either demonstrated or strongly speculated, highlighting the increasing role of globalised trade and international travel as a means for *Pst* urediniospore dispersal.

### Pathogen evolution and adaptation

Prior to 2000, pathogenicity surveys and molecular studies using isolates collected across the main wheat-producing regions in Europe, Australia and America typically reported *Pst* populations were clonal in nature, and that pathotypes exhibited close-relatedness and low genetic variation—predominantly underpinned by single step-wise mutations (Hovmøller et al. 2002, 2016; Enjalbert et al. 2005; Chen 2005; Steele et al. 2001; Chen et al. 2010; Ali et al. 2014a; Hubbard et al. 2015). Such clonally derived *Pst* mutations have caused several severe YR epidemics, due to the ‘breakdown’ of specific wheat *Yr* resistance genes present in large acreages across the agricultural landscape. Notable examples include breakdown of *Yr17* in Northern Europe (Bayles et al. 2000), *Yr27* in Ethiopia (Solh et al. 2012), and *Yr9* in America, the Middle East and the Indian sub-continent (Chen et al. 2010; Singh et al. 2004). Before the year 2000, the only exceptions to such patterns of low *Pst* genetic variation were observed in isolates from the Himalayan (Nepal and Pakistan) and near Himalayan (China) regions, which exhibited high levels of genetic recombination, high ability for sexual reproduction and high genetic diversity (Duan et al. 2010; Mboup et al. 2009; Ali et al. 2014b). These areas were therefore classified the putative centres of *Pst* origin (Ali et al. 2014b). However, the last two decades have seen the emergence of unusual virulence profiles and aggressive strains across the world. The most noteworthy event was the rise of two strains, *PstS1* and *PstS2,* across the USA (Chen et al. 2002; Markell and Milus 2008), Europe (Hovmøller and Justesen 2007) and Australia (Wellings 2007) in the space of just three years in the early 2000s. A global study of pre- and post-2000 *Pst* races combining detailed virulence pathotyping and DNA fingerprinting found that while these while these two strains were genetically similar to each other, they were highly divergent from previous races in their respective geographic regions (Hovmøller et al. 2008). Their rapid spread was thought to be due to their increased aggressiveness (ability to yield more spores and for disease symptoms to occur more quickly) and high-temperature adaptation—which was later demonstrated in the detailed study by Milus et al. (2009). In addition to *PstS1* and *PstS2*, additional atypical occurrences of *Pst* races have since been reported. Enjalbert et al. (2005) demonstrated high levels of genetic divergence between the *Pst* population in northern France and a single clone specific to the South. What was atypical was that this single pathotype was maintained for a long time in this region, despite the presence of gene flow between Northern and Southern *Pst* populations. This isolate was later found to be more closely related to the Central Asian-Mediterranean population (Ali et al. 2014a). Similarly, instances of strong genetic divergence have also been revealed in North Western Europe (Flath and Barthels 2002; Hovmøller and Justesen 2007a). Two groups of highly divergent pathotypes from the ‘old’ North-Western European population exhibited three to four times higher levels of genetic diversity (Hovmøller et al. 2007). In 2011, two novel *Pst* races disrupted the European *Pst* landscape (www.wheatrust.org). Named after the host varieties on which they were first detected, one race was virulent on wheat cv. ‘Warrior’ and the other was virulent on cv. ‘Kranich’. These were later characterised as *PstS7* and *PstS8,* respectively (Ali et al. 2017), and were detected simultaneously across Europe and infected varieties that had exhibited durable adult plant resistance. Both races were distinct from the typical European isolates in that they produced an unusually high number of teliospores (Hubbard et al. 2015; Hovmøller et al. 2016). Additional *Pst* races have been characterised (*PstS10* also known as ‘Warrior (-)’, *PstS4* ‘Triticale aggressive’) and together with the other new genetically diverse *Pst* races, have come to largely dominate within Europe (Ali et al. 2017; Hovmøller et al. 2016; Hubbard et al. 2015). Collectively, these atypical observations, further supported by genetic diversity studies, have led to speculation of an aerial-induced foreign incursion, which would be the first of its kind in Europe since the establishment of *Pst* in Europe during the nineteenth century. Beyond Europe, rapid invasions and the subsequent *Pst* population changes have been responsible for a number of YR epidemics in Central Asia, North and East Africa (Ali et al. 2017).

## Chemical control of yellow rust

Review of global YR epidemics shows most wheat growing regions document recurrent crop losses of 5–10%, with occasional losses of up to 25% (Welling 2011). However, following the global spread of aggressive *Pst* races since 2000, surveys highlight an increase in both the number of countries being significantly hit by such races, and the extent of the losses incurred (Beddow et al. 2015). Indeed, the financial implications of this change in *Pst* race structure estimated that a global average of US$ 158 million was lost annually pre 2000s, compared to US$ 979 million post 2000 (Beddow et al. 2015). Wheat growers have two principal options to protect against the effects of YR on yield: (i) protect their crop with agro-chemicals that limit initial infection and progression of pathogen colonisation, and/or (ii) grow wheat varieties with adequate levels of genetic resistance. Systemic fungicides that are absorbed into the plant became commercially available in the 1980s and have since formed an important part of integrated control measures against YR (Chen 2005). Several products with different modes of action are available for protection against YR (reviewed by Chen and Kang 2017), with timely application a key aspect of an effective fungicide programme. Such an approach has, for example, prevented significant financial losses in periods of severe epidemics in the USA (Line 2002). While fungicide control provides an essential tool in combatting sudden yellow rust epidemics and in situations where growing resistant varieties is not an option, over-dependence on their use comes with negative environmental impacts and notable financial cost to growers. For example, in Australia an estimated A$ 359 million per year is spent on fungicides for YR control (Murray and Brennan 2009). In the mid-to-long term, regular *Pst* exposure to fungicides also increases the risk that *Pst* populations develop resistance to frequently used chemistries. Historically, *Pst* has been classified as being at low-risk of developing fungicide resistance. However, of the three classes of fungicides active against *Pst* (demethylation inhibitors, DMIs; succinate dehydrogenase inhibitors, SDHIs; quinone outside inhibitors, QoIs), *Pst* resistance has evolved against two. Low levels of DHI resistance have been reported, and while high proportions of isolates carrying resistance associated mutations have been reported in some countries (Cook et al. 2021), DHI resistance has so far had limited agronomic-scale significance (Oliver 2014). SDHIs active against rusts have only been introduced relatively recently, giving less time for *Pst* resistance to evolve. Nevertheless, sets of geographically diverse isolates have been identified that carry a mutation homologous to that linked to SDHI resistance in the related rust species *P. pachyrhzi* (Cook et al. 2021). In the face of additional considerations such as changing regulation surrounding permissible chemistries, such evidence has led to the suggestion that the *Pst* risk classification should be upgraded (Oliver 2014), fungicide resistance management practices be considered, and that systematic monitoring for *Pst* fungicide resistance should be implemented (Cook et al. 2021). Lastly, the optimisation of fungicide timing, as well as improved fungicide application technologies, represents areas where additional research and development is required (Carmona et al. 2020).

## Genetic control of yellow rust

More than 300 wheat genomic regions conferring YR resistance have been reported (Rosewarne et al. 2013; Wang and Chen 2017). Of these, ~ 80 are permanently named yellow rust resistance (*Yr*) genes (recently summarised by Jamil et al. 2020). Two main classes of YR resistance (*R*) genes are commonly described. The first is termed ‘all stage resistance’ (or ‘seedling resistance’) and confers qualitative resistance–typically to one or a low number of *Pst* isolates. The second is termed ‘adult plant resistance’ (APR) and confers quantitative or partial resistance. While these *R* gene classifications are useful, additional categories are also used, based on criteria such as phenotypic response (infection type, race specificity, resistance levels), temperature sensitivity, durability, the number of genes involved (monogenic versus polygenic) and the size of gene effect (Chen 2013). One of the issues that comes with defining YR resistance with such a broad range of criteria is the assumptions associated with each of them. For example, APR is typically non-race specific, more durable than seedling resistance and conditioned by genes with minor or partial effect. Nevertheless, some APR genes have been shown to exhibit race specificity, such as *Yr11, Yr12, Yr13* and *Yr14* (Johnson 1992; McIntosh et al. 1995).

### All-stage resistance

Initially expressed at the seedling stage, all-stage resistance extends throughout the growth of the wheat plant and is characterised by a hypersensitive response. It is generally effective against some, but not all, *Pst* races and is therefore also referred to as ‘race-specific resistance’. All-stage resistance is underpinned by the gene-for-gene model, first explored by Flor (1956) in the flax-rust pathosystem, whereby the product of an *R* gene must be recognised by the protein encoded by its corresponding avirulent (*Avr*) gene in the pathogen, with resistance conferred by an incompatible *R-Avr* interaction. This results in a qualitative resistance phenotype that can be easily assessed, historically making it a popular selection criterion in breeding programmes, and more recently, for gene cloning. The majority of catalogued YR *R* genes exhibit this type of phenotype, and many become ineffective against present-day *Pst* races. This type of resistance has commonly been shown to be a short-term strategy for YR control. Indeed, the deployment of varieties with single or low-numbers of all-stage resistance *Yr* genes over large acreages inevitably exerts high selective pressure on the pathogen, forcing it to evolve and mutate until host resistance is broken down, and leading to cycles of ‘boom and bust’ (McDonald and Linde 2002).

### Adult plant resistance

Adult plant resistance (APR) is characterised by slow rusting (a long period of latent infection, small lesion size) (Guo et al. 2008) or partial resistance, typically manifests at the adult plant stage, and has long been established as a durable source of YR resistance. Two notable examples are *Yr18/Lr34/Sr67/Pm38*, extensively deployed in spring wheat cultivars through the international breeding programme at CIMMYT (Singh et al. 2005) and *Yr16*, an APR gene commonly used in early European varieties such as ‘Cappelle Desprez’, a major hub in the European wheat pedigree (Fradgley et al. 2019). While APR is primarily non-race specific, examples of APR specificity to *Pst* races do exist, such as *Yr12* and *Yr13* (Johnson 1992; McIntosh et al. 1995). Such APR race-specificity was initially reported by Johnson (1988) and has recently been observed in Europe following the spread of atypical *Pst* races (Sørensen et al. 2014). For example, while the APR resistance allele conferred by the founder Claire at the QTL *QYr.niab-2D.1* was effective in the UK during the 2015 and 2016 seasons (Bouvet et al. 2021b), it has since broken down (Simon Berry, personal communication). Another example is that of *Yr49*, which was initially found to be non-race specific against all Australian *Pst* isolates, but when tested against Chinese races showed race-specificity (Ellis et al. 2014). These occurrences undermine the durability of APR and puts into question whether this pathotype criteria should be used to describe this type of resistance. It has been suggested that as some APR genes confer resistance against multiple biotrophic pathogens, this characteristic is a good indicator of durability. Examples include *Yr18/Lr34/Sr67/Pm38* (Spielmeyer et al. 2005; Lillemo et al. 2008), *Yr29/Lr46/Sr58/Pm39* (Lagudah, 2011), *Yr30/Lr27/Sr2* (Mago et al. 2011) and *Yr46/Lr67/Sr55/Pm46* (Herrera-Foessel et al. 2014). Interestingly, some of these genes are also associated with traits such as leaf tip necrosis (*Yr18/Lr34/Sr67/Pm38,* Singh et al. 1992; *Yr29/Lr46/Sr58/Pm39*, Rosewarne et al. 2006; *Yr46/Lr67/Sr55/Pm46*, Herrera-Foessel et al. 2014) and pseudo-black chaff (*Yr30/Lr27/Sr2*, Kota et al. 2006). Finally, some APR resistances are more effective at high temperature (usually 25–30 °C), and are termed High Temperature Adult Plant (HTAP) resistance. *Yr36* was initially characterised as HTAP (Uauy et al. 2005), with subsequent studies showing resistance was effective over 25 °C at all growth stages (Fu et al. 2009), and that the lower effective temperature range is 18 °C (Bryant et al. 2014).

## Cloned yellow rust resistance genes

Nucleotide Binding Sequence Leucine Rich Repeat (NBS-LRR) proteins are the most common class of proteins encoded by plant *R* genes, and act predominantly by recognising the effector molecules that pathogens produce to inhibit host defence responses (Jones et al. 2016). To help fight against potential infecting pathogens, plant NLR gene families have radiated and diversified, for example via localised gene duplication or mutation within their LRR domains that bind pathogen effectors (Sarris et al. 2016). Furthermore, some NBS-LRRs contain additional ‘integrated’ domains, the most common of which are kinase and DNA-binding domains (Andersen et al. 2020; Steuernagel et al. 2020), and are thought to be involved in receptor activation or downstream signalling (Sarris et al. 2016). Of the 19 genes conferring all-stage resistance to wheat rusts (yellow rust, stem rust, leaf rust) that have been cloned, 17 encode NBS-LRRs (Table 1). Furthermore, all but two of these 17 NBS-LRRs contain coiled coil (CC) domains towards their N-termini; the exceptions being *Yr7* and the allelic *R* genes *Yr5*/*YrSP*, each of which contains an N-terminus integrated BED zinc finger domain (Marchal et al. 2018) and *Sr60*, which is race-specific but confers a partial resistance phenotype and encodes a protein with two putative kinase domains (Chen et al. 2020). Finally, the broad-spectrum ASR gene *Yr15* encodes a tandem kinase-pseudokinase protein (Klymiuk et al. 2018) similar to that encoded by the barley stem rust resistance gene *Rpg1* (Brueggeman et al. 0.2002), and has recently been shown to be allelic with *YrG303/YrH52* (Klymiuk et al. 2020).

**Table 1:** Cloned wheat rust resistance (*R*) genes.

Cloned YR resistance genes	Original source	Chr	***R*** gene class	NCBI protein accession number	Gene functional annotation	Reference
*Lr1*	*T. aestivum*	5D	ASR	ABS29034	CC-NBS-LRR	Cloutier et al. (2007)
*Lr10*	*T. aestivum*	1A	ASR	AAQ01784	CC-NBS-LRR	Feuillet et al. (2003)
*Lr21*	*Ae. tauschii*	1D	ASR	ACO53397	NBS-LRR	Huang et al. (2003)
*Lr22a*	*Ae. tauschii*	2D	ASR	ARO38244	CC-NBS-LRR	Thind et al. (2017)
*Sr13*	*T. turgidum* ssp. *durum*	6A	ASR	ATE88995	CC-NBS-LRR	Zhang et al. (2017)
*Sr21*	*T. monococcum*	1D	ASR	AVK42833	CC-NBS-LRR	Chen et al. (2018)
*Sr22*	*T. monococcum*	7A	ASR	CUM44200	CC-NBS-LRR	Steuernagel et al. (2016)
*Sr33*	*Ae. tauschii*	1D	ASR	AGQ17384	CC-NBS-LRR	Periyannan et al. (2013)
*Sr35*	*T. monococcum*	3A	ASR	AGP75918	CC-NBS-LRR	Saintenac et al. (2013)
*Sr45*	*Ae. tauschii*	1D	ASR	CUM44213	CC-NBS-LRR	Steuernagel et al. (2016)
*Sr46*	*Ae. tauschii*	2D	ASR	AYV61514	CC-NBS-LRR	Arora et al. (2019)
*Sr50*	*Secale cereale*	1R^†^	ASR	ALO61074	CC-NBS-LRR	Mago et al. (2015)
*Sr60*	*T. monococcum*	5A	ASR	LRRK123	Tandem kinase	Chen et al. (2020)
*SrTA1662*	*Ae. tauschii*	1D	ASR	*Not listed*	CC-NBS-LRR	Arora et al. (2019)
*YrAS2388*	*Ae. tauschii*	4D	ASR	QDW65446	CC-NBS-LRR	Zhang et al. (2019)
*Yr5/YrSP*	*T. spelta album*	2B	ASR	QEQ12705/QEQ12706	BED-NBS-LRR	Marchal et al. (2018)
*Yr7*	*T. aestivum*	2B	ASR	QEQ12704	BED-NBS-LRR	Marchal et al. (2018)
Yr10^*^	*T. aestivum*	1B	ASR	AAG42168	CC-NBS-LRR	Liu et al. (2014)
*Yr15/YrG303/YrH52*	*T. turgidum* ssp. *dicoccoides*	1B	ASR	AXC33067	TKP	Klymiuk et al. (2018)
*Yr18/Lr34*	*T. aestivum*	7D	APR	ACN41354	ABC transporter	Krattinger et al. (2009)
*Yr36*	*T. turgidum* ssp. *dicoccoides*	6B	APR	ACF33187	Kinase-START	Fu et al. (2009)
*Yr46/Lr67*	*T. aestivum*	4D	APR	ALL26331	Hexose transporter	Moore et al. (2015)

*ASR*  all-stage resistance. *APR*  adult plant resistance. *Lr*  leaf rust, *Sr*  stem rust, *Yr*  yellow rust. *TKP*  tandem kinase-pseudokinase. *Chr*.  chromosome.

^†^In bread wheat, the *Sr50* locus from rye has been translocated to chromosome 1D

*See also Yuan et al. (2018), who indicate the CC-NBS-LRR gene identified by Liu et al. (2014) may not be the underlying gene

The ongoing changes and rapid spread of *Pst* populations around the world has led to growing interest in more durable sources of resistance. To date, three adult plant YR resistance genes have been cloned. *Yr36* encodes a protein with a kinase and a START lipid-binding domain (WHEAT KINASE START 1*,* WKS1; Fu et al. 2009), and is thought to regulate reactive oxygen species (ROS) via phosphorylation of the thylakoid ascorbate peroxidase protein, resulting in increased levels of ROS during immunity (Gou et al. 2015). More recently, WKS1 has been shown to phosphorylate a protein component of photosystem II, sbO, resulting in reduced photosynthesis, leaf chlorosis and *Pst* resistance (Wang et al. 2019). *Yr18/Lr34* encodes an ABC transporter (Krattinger et al. 2009) involved in the translocation of abscisic acid (Krattinger et al. 2019) while *Yr46/Lr67* encodes a hexose transporter (Moore et al. 2015).

## Designing yellow rust resistant wheat

Pyramiding multiple resistance genes with additive effects into single genetic backgrounds should help prevent dramatic breakdown of wheat *Pst* field resistance. This first iteration of resistance gene pyramiding was developed using conventional breeding techniques. Indeed, the CIMMYT wheat breeding programme has made extensive use of the ‘*Yr18* complex’ (*Yr18* and at least two to three additional slow-rusting genes), which has provided durable resistance against yellow rust (Singh et al. 2005). Tools to help such approaches are available. These include protocols for the use of diagnostic molecular markers for marker-assisted breeding for many of the cloned resistance genes listed above (https://maswheat.ucdavis.edu/), as well as ‘speed breeding’ methods that include the use of extended day lengths and controlled temperatures to shorten the wheat lifecycle (Watson et al. 2018). Indeed, knowledge of which resistance genes are present within breeders germplasm/released wheat varieties would help prioritise parental lines for future breeding efforts. However, combining numerous unlinked genes via crossing is time-consuming. For example, a recent crossing scheme for the incorporation of 12 resistance genes in a single recurrent background involved 20 generations (Hafeez et al. 2021). Additionally, sources of YR resistance commonly originate from species related to bread wheat (see Table 1), including diploid wheat (e.g. *T. monococcum* and *Aegilops tauschii*) and wild or cultivated tetraploid wheats (*T. turgidum* ssp. *dicoccoides* and *T. turgidum* ssp. *durum*, respectively), resulting in introgression of linked chromosomal regions from the donor progenitor species. Such introgressed regions may have a negative effect on crop performance; for example, while *Sr60* has recently been introduced into bread wheat via the introgression of a small *T. monococcum* segment containing the *R* gene, it nevertheless contains linked *PUROINDOLINE* genes which will affect grain texture (Chen et al. 2020). Furthermore, it can be challenging when crossing germplasm within conventional breeding programmes to maintain the desired resistance gene combinations in the progeny, as the loci are inherited independently. In practice therefore, the combinations of resistance genes deployed by breeders will also depend on the genetic architectures controlling many other agronomically important traits. This means that key resistance loci may be at risk of being used alone, leaving them exposed to be overcome by the pathogen. Such considerations mean development of resistance gene cassettes containing multiple *R* genes could provide a useful breeding tool, providing multiple sources of resistance inherited as a single genetic unit. Assuming their effects will be additive (i.e. show no epistasis), the three cloned APR genes, *Yr18, Yr36* and *Yr46*, possibly combined with one or more ASR genes such as *Yr15*, represent obvious immediate targets. Indeed, a transgene cassette containing four stem rust ASR genes and one APR gene has recently been shown to confer broad-spectrum field resistance (Luo et al. 2021). However, such approaches do not come without their challenges: genetic modification regulations and consumer acceptance remains an important barrier in many parts of the world, relatively low numbers of *Yr* genes have been cloned, and further work is needed to determine how specific genes work in combination within the context of inbred lines and F_1_ hybrids. Towards tackling some of these issues, proposals to generate an *R* gene atlas for the major diseases of wheat have been made (Hafeez et al. 2021). Such concepts would be aided by the systematic identification and monitoring of the corresponding *Pst* effectors and their standing variation across the agricultural environment, and should be extended to identify, characterise and eliminate wheat susceptibility (*S*) genes that act to increase YR susceptibility (e.g. Corredor-Moreno et al. 2021). Underpinning such aims is the availability of new genomic techniques and resources in wheat that complement classical map-based cloning methodologies (recently reviewed by Adamski et al. 2020). For example, candidate gene association mapping using diversity panels of wheat or wheat relatives genotyped via reduced representation sequencing of classes of genes known a priori to be prevalent in disease resistance (such as NBS-LRRs or wall-associated kinases). This method, termed ‘RenSeq’ (Jupe et al. 2013), alongside functional validation via chemical mutagenesis of germplasm containing the functional allele of interest, has been used to identify the wheat stem rust resistance genes *Sr46* and *SrTA1662* (Arora et al. 2019). Such association mapping approaches can be extended to include more representative coverage across the genome, for example using promotor/exome capture arrays (Gardiner et al. 2019) or whole-genome sequencing at low-coverage combined with imputation of SNPs and haplotypes, aided by the use of reference genome assemblies (e.g. for bread wheat: IWGSC, 2018; Walkowiak et al. 2021). Furthermore, the availability of *Pst* genome assemblies (e.g. Cantu et al. 2011, 2013; Zheng et al. 2013; Schwessinger et al. 2018, 2020) and mutant populations (Li et al. 2020), as well as gene expression resources and interrogation tools for both species (e.g. Adams et al. 2021) should help identify and characterise pathogen effectors. Detailed knowledge of the specificity of the recognition interactions between wheat *R* genes and their corresponding *Pst* effectors could be used, for example, to monitor the functionality of each component of *R* stacks, and to design synthetic *R* genes engineered to recognise multiple races (as demonstrated for example by editing of the rice NBS-LRR gene *PikP* to recognise multiple variants of the effector AvrPik from the rice blast pathogen *Magnaporthe oryzae*; De La Concepcion et al. 2019). Similarly, identification of wheat *S* genes would allow their elimination, via marker assisted approaches, mutation breeding or gene editing. Finally, further understanding of the exact developmental stages at which different adult plant resistance genes become effective, how best to deploy these in the agricultural landscape to best protect the crop from infection throughout the key growth stages, and understand which *R* genes exhibit the lowest yield cost, will further help protect wheat against the effects of YR.

## Future perspectives

The wide-ranging spread of new genetically diverse *Pst* races has meant that YR is likely to become an increasing threat to global wheat production, resulting in lower yields and increased financial and environmental costs. Here, we conclude with a series of bullet-point recommendations for future research and development in YR management over the next decade:

### Host genetics

1. Systematic programmes to identify and clone known and novel *R* genes, particularly those conferring adult plant or non-host resistance.
2. Informed design and development of durable *R* gene pyramid combinations, via traditional crossing and/or *R* gene cassettes.
3. Identification and targeted removal of susceptibility (*S*) genes from breeders’ germplasm.

### Monitoring

1. Regional and international networks to rapidly monitor the emergence and spread of *Pst* pathotypes.
2. Field networks to monitor *R* gene effectiveness at regional/international scales.

### Agronomy

1. Regional monitoring for the emergence and spread of fungicide resistances.
2. Innovation in fungicide application technology and crop monitoring to allow more timely, accurate and efficient fungicide application.

Implementation of these recommendations will work best when national programmes are integrated or are coordinated at a regional, or even global, level. Such coordination would require funding over timescales that go beyond that typically available for crop disease resistance research, and might best be best addressed by establishing regional coordination centres. Such networks would need to ensure fast and efficient data release and work closely with the crop breeding industry. Ultimately, the success of advances in integrated YR management approaches will depend on timely communication of information to wheat growers. Therefore, trusted grower-facing networks and sources of information that can rapidly and succinctly inform and advise farmers of threats and best practice within each growing season will become increasingly critical in realising future ambitions to better protect wheat yields from diseases such as YR.

## Abbreviations

APR: Adult plant resistance
DMIs: Demethylation inhibitors
GWAS: Genome-wide association scan
MAS: Marker assisted selection
NLR: Nucleotide-binding leucine-rich repeat
*Pst*: *Puccinia striiformis* Westend f. sp. *tritici*
QTL: Quantitative trait locus
QoIs: Quinone outside inhibitors
SDHIs: Succinate dehydrogenase inhibitors
YR: Yellow rust
